# Dietary Quality, Sleep Quality and Muscle Mass Predicted Frailty among Chinese Postmenopausal Women in Malaysia

**DOI:** 10.3390/ijerph19052565

**Published:** 2022-02-23

**Authors:** Kai Sze Chan, Yoke Mun Chan, Yit Siew Chin, Zalilah Mohd Shariff

**Affiliations:** 1 Department of Dietetics, Faculty of Medicine and Health Sciences, Universiti Putra Malaysia, Seri Kembangan 43400, Selangor, Malaysia; kenszechan@gmail.com; 2 Research Center of Excellence, Nutrition and Non-Communicable Diseases, Universiti Putra Malaysia, Seri Kembangan 43400, Selangor, Malaysia; 3 Malaysian Research Institute on Ageing, Universiti Putra Malaysia, Seri Kembangan 43400, Selangor, Malaysia; 4 Department of Nutrition, Faculty of Medicine and Health Sciences, Universiti Putra Malaysia, Seri Kembangan 43400, Selangor, Malaysia; chinys@upm.edu.my (Y.S.C.); zalilahms@upm.edu.my (Z.M.S.)

**Keywords:** frailty, Chinese postmenopausal women, dietary quality, skeletal muscle mass, sleeping quality

## Abstract

The older adult population is growing faster than any age group, which increases their risk of frailty. Studies conducted among older adult are relatively scarce in Malaysia, especially among Chinese postmenopausal women, who have the longest life expectancy. Thus, the present study aimed to determine the prevalence of frailty and its associated factors among Chinese postmenopausal women. A total of 220 eligible respondents were recruited, with information on sociodemographic background, comorbidities, dietary intake and lifestyle behaviour were obtained using a structured questionnaire, while anthropometry indicators were assessed according to standard protocol. Fasting blood was withdrawn for the analysis of serum 25(OH)D. Multinomial logistic regression was used to determine factors that predict pre-frailty and frailty. Prevalence of pre-frailty and prevalence of frailty were 64.5 and 7.3%, respectively, and most of the respondents presented with weak handgrip strength. Pre-frailty was prevalent among the younger population. Dietary quality was unsatisfactory among the respondents, and the majority of them presented with a high percentage of body fat. An increased dietary quality index (DQI), poor sleep and low muscle mass were factors that contributed to frailty. In conclusion, nutritional factors should be considered in developing health-related policies and programs in reducing and delaying the onset of frailty.

## 1. Introduction

Worldwide, there is an increase in life expectancy, representing one of the key drivers of population ageing in addition to decreased fertility. The pace of population ageing is increasing dramatically, with the elderly population expected to be doubled or tripled in the years 2050 and 2100, respectively [1]. Malaysia is not exempt from the ageing population scenario commonly observed in Asian countries. According to the Department of Statistics Malaysia, approximately 7% of the total population was older people in 2019 [2], and this is expected to reach 14.5% in 2040 [3]. However, this increase in longevity often does not correspond with better functional abilities. Frailty, a recognised geriatric syndrome, is expected to increase with the substantial growth of the elderly population. The prevalence of frailty among the population aged 50 years old and above in 18 European countries was 7.7%, with almost half of the population being pre-frail [4]. Across Asia, the highest prevalence of frailty was reported in Hong Kong and Beijing [5,6,7]. In Malaysia, the prevalence of frailty was 15.9% among the elderly population in a recent study [8]. Individuals with frailty had an increased risk of disability and mortality [9], which deteriorated their health and increased individual and nation medical expenditures [10]. In addition to increased risk of disability, individuals with frailty may also have an increased risk of cardiovascular disease-related morbidity and mortality [11], which signify the need for appropriate preventive measures.

To the best of knowledge, previous studies on frailty in Malaysia focused on the elderly population aged 60 years and above [8,12,13,14,15]. Contrary to popular belief, frailty is not limited to older people [16], and it is indeed an overlooked problem among the middle-aged population. Precursors of frailty tend to arise earlier in the life course, where the antecedents of frailty in late life could have manifested at middle age [16]. Frailty predicts future disability, but it is modifiable, particularly at an early stage [17], suggesting that identifying predictors for frailty and pre-frailty in younger populations might reduce the occurrence of frailty when they are older. Despite the importance of identifying the risk of frailty in middle-aged people, most epidemiological studies assessing frailty have excluded those younger than 65 years, and, as such, little is known about frailty in younger populations [17]. In the absence of appropriate intervention or health policies, the middle-aged population, especially postmenopausal women, is expected to experience a steep increase in the risk of frailty when their age increases. Hence, the present study aimed to investigate the magnitude of frailty among middle-aged and older postmenopausal women and factors associated with frailty among these postmenopausal women in Malaysia.

## 2. Materials and Methods

### 2.1. Study Design and Study Population

This was a cross-sectional study, and a total of 220 community-dwelling postmenopausal Chinese women were recruited from affiliates of senior citizen organisation in Kuala Lumpur and Selangor, Malaysia. Ethical approval was granted (Ethics Committee for Research Involving Human Subjects of Universiti Putra Malaysia; reference number: JKEUPM-2018-056; approved on 31 May 2018), and permission to conduct this study was obtained from the respective affiliates before study enrolment. Eligibility of respondents was limited to women who had menopause naturally for at least five years, while those with medical diagnoses of severe diseases, such as cardiovascular diseases, cancer, stroke, Parkinson disease and impaired liver and renal function, were excluded. Written consent was obtained from the respondents before study commencement.

### 2.2. Assessment of Frailty

The presence of frailty among respondents was ascertained based on the most widely cited phenotype criteria [9], namely, unintentional weight loss, self-reported exhaustion, decreased handgrip strength, low gait speed and low energy expenditure. The presence of weight loss among the respondents was ascertained with the following question “In the last year, have you lost more than 4.5 kg unintentionally (i.e., not due to dieting or exercise)?” Respondents who answered “Yes” and did not report any ongoing weight loss programme were considered to have experienced unintentional weight loss. Exhaustion was assessed using the depression scale of the Centre for Epidemiological Studies (CES-D) [18], which comprises the following questions: “How often in the last week did you feel that everything you did was an effort?” and “How often in the last week did you feel that you could not get going?” Possible responses were on a four-point Likert Scale, which ranged from 0 = rarely or none of the time (<1 day), 1 = some or a little of the time (1–2 days), 2 = a moderate amount of the time (3–4 days), to 3 = most of the time. Respondents who chose “2” or “3” to any two questions were considered to self-report exhaustion. The handgrip strength of respondents was measured using a routinely calibrated dynamometer on their dominant hands, following a standard protocol. An average reading of handgrip strength was taken from two measurements and compared to the existing cut-off value [9], as stratified by the body mass index (BMI) of respondents.

On the other hand, time taken to complete a 4.5 m distance walk was used to determine the gait speed of the respondents. Respondents with a walking time equal to or more than the recommended cut-off value (for height ≤ 159 cm, cut-off value ≥ 7 s; for height > 159 cm, cut-off value ≥ 6 s) were considered to possess a slow gait speed. The level of physical activity of respondents was assessed with the Global Physical Activity Questionnaire (GPAQ) [19], which quantifies the physical activity level of respondents of various aspects, such as work, travel, recreation, and sedentary activities in the past seven days, with an energy expenditure of less than 270 kcal/week considered as a low physical activity level. Respondents were classified as non-frail if they did not present with any of the five criteria; respondents presenting with one to two criteria were considered pre-frail, while respondents who fulfilled at least three criteria were considered frail.

### 2.3. Sociodemographic Background

Information on sociodemographic background, such as age, years of education, marital status and monthly household income, was obtained. The monthly household income (in Ringgit Malaysia (RM) 3.98 = USD 1 at the time of data collection) was categorised into low (<RM 2300, or USD 588), medium (RM 2300–5599 or USD 588–1407) and high (>RM 5600 or USD 1407) based on the Malaysian Economic Planning Unit [20].

### 2.4. Dietary Quality Index (DQI)

All respondents were requested to recall their habitual food intake for the past month using a validated semi-quantitative food frequency questionnaire (sFFQ) adapted from the Malaysian Adult Nutrition Survey 2014 [21]. The sFFQ consists of 165 food items and is categorised into 13 food groups. The amount of food and beverages consumed by the respondents in household measures (bowls, cups, and plates) was converted into metric quantities (in gram) and analysed further using NutritionistPro^®^ software (Axxya System, 2008). The dietary quality of respondents was assessed with the Healthy Eating Index for Malaysians (HEI-M), which was developed and validated among the Malaysian population [22]. The HEI-M consists of nine components, whereby the seven components, namely, cereal and grains, fruits, vegetables, meat, egg and poultry, fish, legumes and milk and dairy products, assessed the dietary adherence of respondents on the Malaysian Dietary Guidelines (MDG) 2010, while the other two components were reported in percentage of total fat consumption and sodium intake [23]. The score of each component ranges from 0 (lack of compliance) to 10 (full compliance), and the score was calculated proportionately for in-between responses. The overall DQI for the respondent was determined by adding up the scores, and a composite score was computed with the following formula: (total score of 9 components/9 × 10) × 100%. Scores from all components are summed up to yield the diet quality, ranging from 0 to 100, with a higher score indicating a good diet quality.

### 2.5. Lifestyle Behaviour

Sedentary behaviour and sleeping quality of respondents were assessed using the Global Physical Activity Questionnaire (GPAQ) [19] and the 19 self-rated Pittsburgh Sleep Quality Index (PSQI) questionnaires, respectively [24]. Permission to use the sleeping quality instrument was obtained before study commencement. Besides an individual’s perception of sleep quality, respondents were assessed on the duration of sleep, habitual sleep efficiency, use of sleep medication, presence of sleep latency (defined as duration to fall asleep), sleep disturbances (defined as any reason that may affect the respondent’s sleep) and daytime dysfunction to allow for the determination of sleep quality. Each component was weighted equally on a 3-point Likert scale, and a score of “0” indicated no difficulty, while a score of “3” indicated severe difficulty. The score from each component was summed to yield the sleeping quality index score, which can range from 0 (no difficulty) to 21 (severe difficulty in all areas) [24].

### 2.6. Anthropometry Measurements

Anthropometry measurements (weight, height and percentage of body fat (PBF)) of the respondents were performed using calibrated instruments. The body weight and height of respondents were assessed with TANITA Digital Weight Scale HD306 (TANITA Corporation, Arlington Heights, IL, USA) to the nearest 0.1 kg and SECA Stadiometer SE217 (SECA, Hamburg, Germany) to the nearest 0.1 cm, respectively. The body mass index of respondents was computed as weight/height^2^ and categorised into four categories: underweight, normal, overweight and obese [25]. The body fat percentage was assessed with the OMRON body fat monitor HBF-306 (Omron Matsusaka Co. Ltd., Matsusaka, Japan) with up to 0.1% accuracy, while the skeletal muscle mass (SMM) of respondents was calculated based on the following formula: SMM (kg) = 0.244 × body weight (kg) + 7.8 × height (m) − 0.098 × age + 6.6 × sex (female = 0) + race (Asian = −1.6) − 3.3 [26]. Presence of sarcopenia was confirmed according to criteria of the Asian Working Group for Sarcopenia (AWGS 2019), namely, respondents with SMM/square of height ≤ 5.7 kg/m^2^ and either low gait speed (walking speed < 1.0 m/s) or low muscle strength (handgrip strength < 18 kg) [27].

### 2.7. Serum Vitamin D

Fasting venous blood was drawn from participants on the morning after a minimum of 8 h of overnight fasting. The specimen was sent to a commercial lab for analysis. Serum vitamin D (25(OH)D) was measured using ADVIA Centaur Vitamin D Total assay with the analytical measuring range between 4.2 to 150 ng/mL (10.5 to 375 nmol/L). Vitamin D status of respondents was classified into three subgroups according to the recommendation by the Institute of Medicine [28].

### 2.8. Charlson Comorbidity Index

The presence of comorbidity was self-reported by respondents, and the Charlson comorbidity index (CCI) was used to quantify the respondents’ comorbid disease burden. The CCI assigned weight for each disease, and the total score was calculated by adding the weights [29]. The Charlson comorbidity index has been used extensively in the medical literature for comorbidity assessment, and it is universally used to predict short- and long-term mortality [30].

### 2.9. Statistical Analysis

The data were analysed using IBM SPSS Statistics 22.0 (SPSS Inc., Chicago, IL, USA). An independent-sample *t*-test was used to determine the mean differences between respondents who were non-frail and at risk of frailty. Pearson chi square (χ^2^) was used to determine the associations between potential factors and risk of frailty. Multinomial logistic regression analysis was performed to determine the odds ratios of factors affecting the risk of frailty among respondents, whereby variables with significance level at *p* < 0.25 at univariate logistic regression analysis were selected into the final model [31]. Binary logistic regression analysis was also performed to determine the odd ratios of factors affecting the diagnostic components of frailty among respondents. The statistical significance level was set at *p* < 0.05.

## 3. Results

### 3.1. Prevalence of Frailty and Distribution of Frailty Components

Prevalence of pre-frailty and prevalence of frailty were 64.5 and 7.3%, respectively (Table 1), with approximately three out of four respondents presented with at least one component of frailty. Slightly more than half of the respondents had weak handgrip strength, followed by another quarter with a low physical activity level. On the other hand, unintentional weight loss was not common, with less than 6% of the respondents self-reporting this issue. A significantly higher number of older respondents (aged 65 years old and above) were pre-frail and frail (χ^2^ = 8.269, *p* < 0.05), presented with more frailty components (χ^2^ = 10.775, *p* < 0.05) and had low grip strength (χ^2^ =4.398, *p* < 0.05) and low gait speed (χ^2^ = 6.241, *p* < 0.05) as compared to their younger counterparts. On the other hand, despite only 2% being frail, more than 60% of the younger respondents were pre-frail.

### 3.2. Characteristics of Respondents

Table 2 shows the distribution of respondents by frailty status as stratified by sociodemographic background, DQI, lifestyle factors, anthropometry indicators and 25(OH)D levels. The mean age of respondents was 66 ± 7 years, and the majority were married and had formal education, with a mean year of education of eight years. The respondents had either a low or a middle household income, while only approximately 20% had a high household income. Dietary quality among the respondents was unsatisfactory, with a mean DQI score slightly higher than 60 out of a possible score of 100. Approximately 87% of the respondents required an improved diet, with another 11% having poor dietary quality. More than half of the respondents were poor sleepers. In addition, despite only a quarter of the respondents being physically inactive (as shown in Table 1), the prevalence of sedentarism was high, with respondents displaying approximately four hours of sedentary behaviour daily.

Regarding anthropometric measurement, more than half of the respondents had a normal body weight but with a high PBF. The mean SMM of respondents was approximately 15 kg, with approximately 20% of the female having low SMM, and 12% were sarcopenic. Overall, the mean 25(OH)D level was approximately 38 nmol/L, with more than 80% of the respondents being either deficient or insufficient in vitamin D.

As the total number of respondents with frailty was small, respondents with pre-frailty and frailty were combined into one group, re-labelled as at risk of frailty (characterised by respondents presenting with at least one component for diagnosing frailty). Approximately one out of three respondents aged less than 65 years old was at risk of frailty. Respondents at risk of frailty were significantly older (t = −2.100, *p* = 0.037), had a longer duration of menopause (t = −2.809, *p* = 0.006) and received fewer years of education (t = 0.928, *p* = 0.045). Despite the means, SMM was comparable between non-frail and frail respondents. All non-frail respondents were not sarcopenic, while approximately one in five frail respondents was sarcopenic (χ^2^ = 10.543, *p* = 0.001). Likewise, the two groups had comparable characteristics.

### 3.3. Factors Contributing to the Risk of Frailty

Table 3 demonstrates the result of a multinomial logistic regression model regarding the associations of frailty with its potential risk or protecting factors. Increased age (OR = 1.155, CI = 1.059–1.259, *p* = 0.001), sedentary behaviour (OR = 1.004, CI = 1.001–1.008, *p* = 0.013) and poor sleep quality (OR = 1.262, CI = 1.085–1.469, *p* = 0.003) were associated with increased risk of frailty, while age less than 65 years old (OR = 0.134, CI = 0.028–0.639, *p* = 0.012) and good sleep quality (OR = 0.071, CI = 0.009–0.572, *p* = 0.013) acted as protective factors. Multivariate logistic regression showed that year of education (OR = 0.920, CI = 0.846–0.999, *p* = 0.047), DQI (OR = 1.045, CI = 1.004–1.087, *p* = 0.030), BMI (OR = 1.269, CI = 1.042–1.547, *p* = 0.018) and SMM (OR = 0.750, CI = 0.578–0.971, *p* = 0.029) contributed significantly to pre-frailty, while an increased DQI (OR = 1.112, CI = 1.025–1.207, *p* = 0.011) was associated with an increased risk for frailty, and being a good sleeper (OR = 0.057, CI = 0.003–0.946, *p* = 0.046) and having adequate muscle mass (OR = 0.068, CI = 0.006–0.796, *p* = 0.032) were protective factors for frailty.

As depicted in Table 4, it is worth noting that risk factors for the components or criteria of frailty vary. While increased age (slow gait speed, *p* < 0.001; weak grip strength, *p* = 0.002), being 65 years old and above (slow gait speed, *p* = 0.010; weak grip strength, *p* = 0.026) and reduced years of education (slow gait speed, *p* = 0.008; weak grip strength, *p* = 0.001) increased the risk of slow gait speed and weak grip strength, respondents with a better dietary quality index were at risk of unintentional weight loss (*p* = 0.040). Respondents with increased CCI were associated with a higher risk of having slow gait speed (multivariable-adjusted OR: 1.945, CI: 1.108–3.416, *p* = 0.021). Compared with their counterparts with better sleep quality, respondents with poor sleep quality had a fivefold higher risk of slow gait speed (multivariable-adjusted OR: 5.406, 95% CI: 1.807–16.172, *p* = 0.003) and approximately threefold higher risk of self-reported exhaustion (multivariable-adjusted OR: 2.996, 95% CI: 1.293–6.941, *p* = 0.010). An increased BMI was associated with a higher risk of weak grip strength (*p* = 0.028), while being underweight increased the risk of unintentional weight loss of the respondents by more than seven times (multivariable-adjusted OR: 7.562, 95% CI: 1.199–47.714, *p* = 0.031).

## 4. Discussion

The prevalence of frailty found in this cohort of postmenopausal women was comparable to that observed in earlier studies in Europe [4], USA [9], Korea [5] and Japan [6] but lower than that noted in other studies in China [7] and Singapore [32]. On the other hand, the prevalence of frailty in this study was comparable to that observed by Badrasawi et al. [16] but lower than that noted in other local studies [8,14,15]. Two-thirds of the participants were pre-frail, which is a higher fraction than that of studies in Europe [4], Brazil [33], Korea [5] and Japan [6] but lower than that of a previous local study [8]. The discrepancies of the prevalence of frailty may be attributed to the different approaches used to diagnose frailty, the different target populations and settings and the sample size of the studies. The most commonly observed feature of weak handgrip strength among the respondents was supported by another study [34]. The prevalence of weak handgrip strength was comparable to that observed in several local studies [13,15], despite being higher than that in another study [35]. On the other hand, the most prevalent component reported in Europe [4], Brazil [33] and Korea [5] was self-reported exhaustion. The finding is not unexpected, as Asians have lower muscle mass or experience a more rapid decline in muscle strength and physical function, leading to weaker handgrip strength [36].

Several factors significantly contributed to pre-frailty, namely, year of education, DQI, BMI and SMM, while DQI, being a good sleeper and having adequate muscle mass significantly contributed to the assessment of frailty. Among the sociodemographic factors, age and being younger than 65 years old significantly contributed to the risk of frailty in the univariate model. The risk of frailty increased with age, which has been proven in other studies [7,35]. Increased age may lead to loss of muscle mass [37] and muscle strength, subsequently leading to a decrease in gait speed and handgrip strength [27], which supports the result of the present study that an increase in age is also associated with slow gait speed and weak handgrip strength.

It is worth noting that pre-frailty is prevalent among respondents below 65 years of age, which, without proper interventions, places them at increased risk of frailty as they age. Appropriate intervention and strategies should be formulated to delay the onset of frailty among these younger individuals. On the other hand, years of education, as well as its components, namely, gait speed and weak handgrip strength, was a protective factor for pre-frailty, which is in accordance with the results of da Alexandre et al. [38] and a recent study in Korea in which elderly people with lower education had poorer handgrip strength [39]. Higher education may act as a mediator that allows an individual to access a better healthcare system; promotes the adoption of healthy behaviours, such as healthy eating and being physically active; and lowers health risk behaviour, such as smoking and consuming alcohol [38], thereby attenuating the risk of frailty and its components. Previous local studies confirmed that individuals with a lower education level had 6.5 times the odds of experiencing unmet needs compared to more highly educated individuals [40] with a lower dietary quality [41]. We failed to find a significant association between age and the risk of frailty in the multivariate model. We do not have an explanation for this, but it could be attributed to factors such as body weight status and social factors, which masked the association of stronger predictors with the risk of frailty and, thus, led to stronger predictors being subsumed [42].

An interesting finding in the present study is that higher DQI increased the risk of pre-frailty and frailty, which is in contrast with the findings of other studies [43,44,45]. The possible explanation for the inconsistent findings may be attributed to different dietary profiles that could produce a similar score; for example, a DQI of 50 can be produced from several different dietary combinations [46]. In addition, the current dietary recommendation may be unable to fit into the daily dietary requirements of postmenopausal women [47]. Development or modification of the dietary recommendation for postmenopausal women should be considered in order to improve the health status of this population. In addition, the time frame of assessing the dietary pattern was for the past one month, which may not represent the respondents’ habitual dietary patterns, or respondents may have alerted their habitual dietary patterns. Cohort or intervention studies would delineate the effect of dietary quality on the risk of frailty in future studies.

In terms of anthropometry indicators, the contribution of BMI to the risk of pre-frailty was not unexpected. Higher BMI was consistently reported to increase the risk of frailty [13,35,48], which may be attributed to the release of resistin by adipocytes due to excessive fat storage [49], resulting in activation of the inflammation process and immune system [50]. Reduced BMI and underweight have been proposed as risk factors for frailty [51], but they were absent in the present study. The relationship between BMI and risk of frailty was proposed to have a U-shaped curve, as either being underweight or overweight increased the risk of frailty [51]. BMI is a surrogate measure of body weight status but fails to reflect body composition (to differentiate between fat mass and fat-free mass). Thus, the measurement of fat mass and muscle mass should be considered when assessing the relationship between BMI and risk of frailty in future studies.

Reduced SMM contributed to a higher risk of pre-frailty and having adequate muscle mass was a protective factor for frailty, echoing the results of recent studies [5,8], wherein a reduction of five units n muscle density was associated with a 20% increase in the risk of frailty. The decrease in SMM may be accompanied by a loss of muscle strength [27], affecting the individual’s handgrip strength and walking speed. However, as frailty and sarcopenia overlap each other [27] and the potential mediating effect of sarcopenia on the outcome of frailty [52], it is difficult to delineate the actual relationship between sarcopenia and the risk of frailty. More extensive work is warranted to explore the potential relationship.

The relationship between 25(OH)D and frailty had long been evident. Recent studies showed that a sufficient level of 25(OH)D is a protective factor for frailty among the older population [5,53,54]. However, the present study failed to demonstrate the relationship between vitamin D and frailty. The possible mechanism is that 25(OH)D receptors were present in several organs and tissues, including muscle tissue. Thus, decreasing age will lead to the deterioration of muscle strength [55]. Discrepancies were observed to exist in another study [56], and, as such, it remains unclear as to whether 25(OH)D is among the factors that cause the development of a frailty phenotype or the consequences of frailty [55]. In short, the relationship between 25(OH)D and the risk of frailty in the present study should be interpreted with caution, as the present study had a small number of respondents with frailty, thus possibly affecting the statistical significance of the relationship.

Being a good sleeper reduced the risk of frailty, which is supported by a recent meta-analysis [57]. It was suggested that poor sleep quality is associated with a high level of inflammatory adipokines [57], which could lead to a catabolic process and the development of sarcopenia [58], thus increasing the risk of frailty. In the present study, poor sleep quality and poor sleepers were both associated with slow gait speed, one of the diagnostic criteria for both sarcopenia and frailty, and this result is in accordance with that of Ershley [58], suggesting the possible influence of poor sleep quality on a higher risk of frailty.

Several limitations should be considered in the present study. Firstly, the present study was a cross-sectional study, making it difficult to determine the cause–effect relationship between factors and the risk of frailty. Secondly, the population involved in the present study were only Chinese postmenopausal women. Thus, it may not be possible to generalise this result to other populations. Thirdly, the diagnostic criteria for frailty were developed based on the Western population, which might be inappropriate for Asians. More prospective cohort studies are needed to delineate the determinants of frailty, and different populations should be assessed to enable the result to be generalised. The development of a cut-off value for the Asian population in diagnosing frailty is recommended.

## 5. Conclusions

In conclusion, approximately two in three postmenopausal women were pre-frail, while 7% were frail, with weak handgrip strength as the most common presenting feature. The present study also revealed that risk of frailty might present as early as middle age, as one out of three postmenopausal women presented with at least one frailty component. Several proposed variables significantly contributed to pre-frailty, namely, year of education, DQI, BMI and SMM, while DQI, good sleeper and adequate muscle mass significantly contributed to the assessment of frailty. The present study emphasises the importance of body weight and body composition management among postmenopausal women, as increased BMI and low SMM could contribute to the risk of frailty. The importance of good sleep quality in reducing the risk of frailty is also highlighted. In light of the expected health and social impacts of frailty on individuals and nations, more work is needed to improve the intervention planning and policy development related to frailty, especially among younger populations.

## Figures and Tables

**Table 1:** Prevalence of frailty and its components of the respondents (*n* = 220).

Characteristics	*n* (%)			*p*-Value
Frailty phenotype	<65 years old	≥65 years old	Total	0.016 *
Non-frail	32 (35.2)	30 (23.3)	62 (28.2)	
Pre-frail	57 (62.6)	85 (65.9)	142 (64.5)	
Frail	2 (2.2)	14 (10.8)	16 (7.3)	
Number of frailty components present				0.029 *
0	32 (35.2)	30 (23.3)	62 (28.2)	
1	40 (44.0)	49 (38.0)	89 (40.5)	
2	17 (18.7)	36 (27.9)	53 (24.1)	
3	2 (2.1)	11 (8.5)	13 (5.9)	
4	0 (0.0)	3 (2.3)	3 (1.4)	
5	0 (0.0)	0 (0.0)	0 (0.0)	
Distribution of frailty components				
Weak handgrip strength	39 (34.2)	75 (65.8)	114 (51.8)	0.036 *
Low physical activity level	19 (33.9)	37 (66.1)	56 (25.5)	0.250
Self-reported exhaustion	12 (34.3)	23 (65.7)	35 (15.9)	0.459
Slow gait speed	5 (17.9)	23 (82.1)	28 (12.7)	0.012 *
Unintentional weight loss	5 (38.5)	8 (61.5)	13 (5.9)	1.000

* Significant at *p* < 0.05, two-tailed.

**Table 2:** Distribution of characteristics of respondents according to frailty status (*n* = 220).

Characteristics	Non-Frailty (*n* = 62)	Risk of Frailty (*n* = 158)	Total	*p*-Value
Age (year)	64.98 ± 5.65	67.05 ± 6.89	66.47 ± 6.62	0.037 **
<65 years old	32 (51.6)	59 (37.3)	91 (41.4)	0.075
≥65 years old	30 (48.4)	99 (62.7)	129 (58.6)	
Duration of menopause (year)	13.98 ± 6.36	16.89 ± 8.13	16.07 ± 7.77	0.006 **
Marital status				
Single/divorced	16 (25.8)	33 (20.9)	49 (22.3)	0.543
Married	46 (74.2)	125 (79.1)	171 (77.7)	
Year of education (years) ^a^	9.01 ± 4.39	7.62 ± 4.66	8.01 ± 4.62	0.045 *
No formal education	5 (8.1)	22 (13.9)	27 (12.3)	0.335
With formal education	57 (91.9)	136 (86.1)	193 (87.7)	
Monthly household income ^b^				
<RM 2300	20 (32.3)	76 (48.1)	96 (43.6)	0.101
RM 2300–5599	27 (43.5)	54 (37.2)	81 (36.8)	
≥RM 5600	15 (24.2)	28 (14.7)	43 (19.5)	
DQI	59.66 ± 8.54	61.68 ± 9.24	61.11 ± 9.08	0.139
Poor (<50.00%) Needs improvement (50.01–80.00%) Good (>80.01%)	9 (14.5) 53 (85.5) 0 (0.0)	15 (9.5) 139 (88.0) 3 (2.5)	24 (10.9) 192 (87.3) 3 (1.4)	-
Sedentary time (min)	224.76 ± 122.81	234.53 ± 148.85	231.77 ± 141.79	0.647
Overall sleeping quality	5.10 ± 3.10	6.06 ± 3.56	5.79 ± 3.45	0.063
Poor sleeper	32 (51.6)	93 (58.9)	125 (56.8)	0.409
Good sleeper	30 (48.4)	65 (41.1)	95 (43.2)	
Body weight (kg)	57.83 ± 7.06	58.15 ± 11.35	58.06 ± 10.31	0.803
BMI (kg/m^2^)	23.88 ± 2.77	24.72 ± 4.73	24.48 ± 4.28	0.107
Underweight	2 (3.2)	8 (5.1)	10 (4.5)	-
Normal weight	37 (59.7)	88 (55.7)	125 (56.8)	
Overweight	23 (37.1)	46 (29.1)	69 (31.4)	
Obese	0 (0.0)	16 (10.1)	16 (7.3)	
PBF (%)	34.74 ± 4.19	35.30 ± 5.47	35.14 ± 5.13	0.465
SMM (kg)	14.98 ± 2.17	14.68 ± 3.03	14.77 ± 2.81	0.474
Low muscle mass (kg/m^2^)	9 (14.5)	34 (21.5)	43 (19.5)	0.322
Adequate muscle mass (kg/m^2^)	53 (85.5)	124 (78.5)	177 (80.5)	
Sarcopenia				
Present	0 (0.0)	27 (17.1)	27 (12.3)	0.001 **
Absent	62 (100.0)	131 (82.9)	193 (87.7)	
Serum vitamin D ^c^ (nmol/L)	36.46 ± 13.87	37.92 ± 14.52	37.51 ± 14.32	0.501
Deficiency (<30 nmol/L)	23 (37.7)	48 (30.8)	71 (32.7)	0.419
Insufficiency (30–49 nmol/L)	26 (42.6)	82 (52.6)	108 (49.8)	
Adequate (≥50 nmol/L)	12 (19.7)	26 (16.6)	38 (17.5)	
CCI	0.45 ± 0.56	0.60 ± 0.73	0.56 ± 0.69	0.107

* Significant at *p* < 0.05, two-tailed. ** Significant at *p* < 0.01, two-tailed. ^a^ Based on 218 of respondents; ^b^ household/personal income range as per The Malaysian Economic Planning Unit (Prime Minister’s Department, Economic Planning Unit, 2016) (Conversion rate, RM3.98 = USD 1 at the time of data collection.); ^c^ based on 217 respondents.

**Table 3:** Multinomial logistic regression between variables and frailty.

Variables	Univariate Logistic Regression				Multivariate Logistic Regression			
	Pre-Frailty		Frailty		Pre-Frailty		Frailty	
	Odds Ratio	CI	Odds Ratio	CI	Odds Ratio	CI	Odds Ratio	CI
Age	NS	NS	1.155 **	1.059–1.259	NS	NS	NS	NS
<65 years old	NS	NS	0.134 *	0.028–0.639	NS	NS	NS	NS
≥65 years old	1		1		1		1	
Marital status								
Single/divorced	NS	NS	NS	NS				
Married	1		1					
Year of education	NS	NS	NS	NS	0.920 *	0.846–0.999	NS	NS
CCI	NS	NS	NS	NS	NS	NS	NS	NS
DQI	NS	NS	NS	NS	1.045 *	1.004–1.087	1.112 *	1.025–1.207
Sedentary behaviour	NS	NS	1.004 *	1.001–1.008	NS	NS	NS	NS
Sleep quality	NS	NS	1.262 **	1.085–1.469	NS	NS	NS	NS
Good sleeper	NS	NS	0.071 *	0.009–0.572	NS	NS	0.057 *	0.003–0.946
Poor sleeper	1		1		1		1	
BMI	NS	NS	NS	NS	1.269 *	1.042–1.547	1.272	0.866–1.868
PBF	NS	NS	NS	NS	NS	NS	NS	NS
SMM	NS	NS	NS	NS	0.750 *	0.578–0.971	NS	NS
Low muscle mass	1		1		1		1	
Adequate muscle mass	NS	NS	0.218 *	0.065–0.735	NS	NS	0.068 *	0.006–0.796
Serum vit D level	NS	NS	NS	NS	NS	NS	NS	NS
Deficiency	1		1		1		1	
Insufficiency	NS	NS	NS	NS	NS	NS	NS	NS
Adequate	NS	NS	NS	NS	NS	NS	NS	NS

* Significant at *p* < 0.05; ** significant at *p* < 0.01; NS = not significant.

**Table 4:** Univariate logistic regression between selected variables and components of frailty (*n* = 220).

Characteristics	Frailty Components				
	Unintentional Weight Loss	Self-Reported Exhausting	Low Energy Expenditure	Slow Gait Speed	Weak Grip Strength
Age	NS	NS	NS	1.130 ** (1.060–1.204)	1.071 ** (1.026–1.117)
<65 years old	1	1	1	1	1
≥65 years old	NS	NS	NS	3.732 * (1.362–10.227)	1.852 * (1.076–3.187)
Years of education	NS	NS	NS	0.885 ** (0.809–0.969)	0.904 ** (0.851–0.961)
CCI	NS	NS	NS	1.945 * (1.108–3.416)	NS
DQI	1.069 * (1.003–1.139)	NS	NS	NS	NS
Sedentary behaviour (min)	NS	NS	NS	NS	NS
Sleep quality	NS	NS	NS	1.181 ** (1.061–1.314)	NS
Good sleeper	1	1	1	1	1
Poor sleeper	NS	2.996 * (1.293–6.941)	NS	5.406 ** (1.807–16.172)	NS
BMI	NS	NS	NS	NS	1.080 * (1.008–1.156)
Underweight Normal weight Overweight Obese	7.562 * (1.199–47.714) 1 NS NS	NS 1 NS NS	NS 1 NS NS	NS 1 NS NS	NS 1 NS -
SMM	NS	NS	NS	NS	NS
Adequate muscle mass	1	1	1	1	1
Low muscle mass	NS	NS	NS	NS	NS
Sarcopenia					
Present	NS	NS	NS	NS	-
Absent	1	1	1	1	
PBF	NS	NS	NS	NS	NS
Serum vit D level	NS	NS	NS	NS	NS
Deficiency	NS	NS	NS	NS	NS
Insufficiency	NS	NS	NS	NS	NS
Adequate	1	1	1	1	1

Data are presented as odd ratios (95% confidence level); * significant at *p* < 0.05; ** significant at *p* < 0.01; NS = not significant.

## Data Availability

Data is contained within the article or supplementary material.
